# Constitutively Active Acetylcholine-Dependent Potassium Current Increases Atrial Defibrillation Threshold by Favoring Post-Shock Re-Initiation

**DOI:** 10.1038/srep15187

**Published:** 2015-10-21

**Authors:** Brian O. Bingen, Saïd F. A. Askar, Zeinab Neshati, Iolanda Feola, Alexander V. Panfilov, Antoine A. F. de Vries, Daniël A. Pijnappels

**Affiliations:** 1 Laboratory of Experimental Cardiology, Department of Cardiology, Leiden University Medical Center, Leiden, the Netherlands; 2 Department of Physics and Astronomy, Ghent University, Ghent, Belgium

## Abstract

Electrical cardioversion (ECV), a mainstay in atrial fibrillation (AF) treatment, is unsuccessful in up to 10–20% of patients. An important aspect of the remodeling process caused by AF is the constitutive activition of the atrium-specific acetylcholine-dependent potassium current (I_K,ACh_ → I_K,ACh-c_), which is associated with ECV failure. This study investigated the role of I_K,ACh-c_ in ECV failure and setting the atrial defibrillation threshold (aDFT) in optically mapped neonatal rat cardiomyocyte monolayers. AF was induced by burst pacing followed by application of biphasic shocks of 25–100 V to determine aDFT. Blocking I_K,ACh-c_ by tertiapin significantly decreased DFT, which correlated with a significant increase in wavelength during reentry. Genetic knockdown experiments, using lentiviral vectors encoding a *Kcnj5*-specific shRNA to modulate I_K,ACh-c_, yielded similar results. Mechanistically, failed ECV was attributed to incomplete phase singularity (PS) removal or reemergence of PSs (*i.e.* re-initiation) through unidirectional propagation of shock-induced action potentials. Re-initiation occurred at significantly higher voltages than incomplete PS-removal and was inhibited by I_K,ACh-c_ blockade. Whole-heart mapping confirmed our findings showing a 60% increase in ECV success rate after I_K,ACh-c_ blockade. This study provides new mechanistic insight into failing ECV of AF and identifies I_K,ACh-c_ as possible atrium-specific target to increase ECV effectiveness, while decreasing its harmfulness.

Atrial fibrillation (AF) is the most common cardiac arrhythmia in humans^1^. Over time, AF usually progresses from paroxysmal, to persistent and finally to permanent AF, partly due to the effect of AF on the arrhythmogenic substrate and/or progression of underlying structural heart disease^2^,^3^. While short self-terminating episodes of AF may remain asymptomatic, the occurrence of longer episodes is associated with morbidity and mortality^2^,^4^,^5^,^6^. Hence, it is important to quickly convert AF to sinus rhythm in order to ameliorate symptoms and to prevent complications, but also to reduce AF-induced atrial remodeling leading to further AF progression. Consequently, termination of AF by electrical cardioversion (ECV) remains the mainstay of acute AF treatment^7^,^8^.

While progression of AF increases the need for ECV, it is also an independent predictor of ECV failure^9^,^10^. ECV may fail to convert AF to sinus rhythm in as much as 10–20% of patients, although the rate of success also depends on the energy applied^9^,^11^. However, perpetually increasing the energy of ECV in case of failure is undesirable, as this is associated with a progressive tendency for adverse events^12^,^13^. To improve acute AF treatment it is essential to increase the effectiveness of ECV and decrease the energy required for successful cardioversion, *i.e.* the atrial defibrillation threshold (aDFT).

A major aspect of the electrophysiological remodeling process associated with AF progression is the shortening of repolarization caused by (among other factors such as decreased L-type calcium current and increased inward rectifier potassium current) constitutive activity of the acetylcholine-dependent potassium current (I_K,ACh_ → I_K,ACh-c_)^14^,^15^,^16^,^17^. Remodeling of I_K,ACh-c_ may therefore play a pivotal role in the diminishing success of ECV with AF progression. I_K,ACh_ is mediated by the Kir3.x family of potassium channels, which are expressed in the atria, but not in the ventricles^18^. As anti-arrhythmic drugs targeting ion channels shared between atria and ventricles have been shown to increase the burden of ventricular arrhythmias^19^,^20^, modulation of I_K,ACh-c_ (as a consequence of its atrial specificity) might provide the advantage of decreasing aDFT without risking alteration of ventricular electrophysiology. However, whether I_K,ACh-c_ affects the ECV success rate and, if so, by which mechanism, remains to be elucidated. Therefore the effects of I_K,ACh-c_ on ECV success rate in a 2D model of reentrant atrial tachyarrhythmias were investigated. Complementary experiments were performed in a whole-heart AF model. Selective inhibition of Kir3.x activity was accomplished using tertiapin or by lentiviral vector (LV)-mediated RNA interference (RNAi).

## Results

### I_K,ACh-c_ blockade decreases aDFT

**Figure 1:**
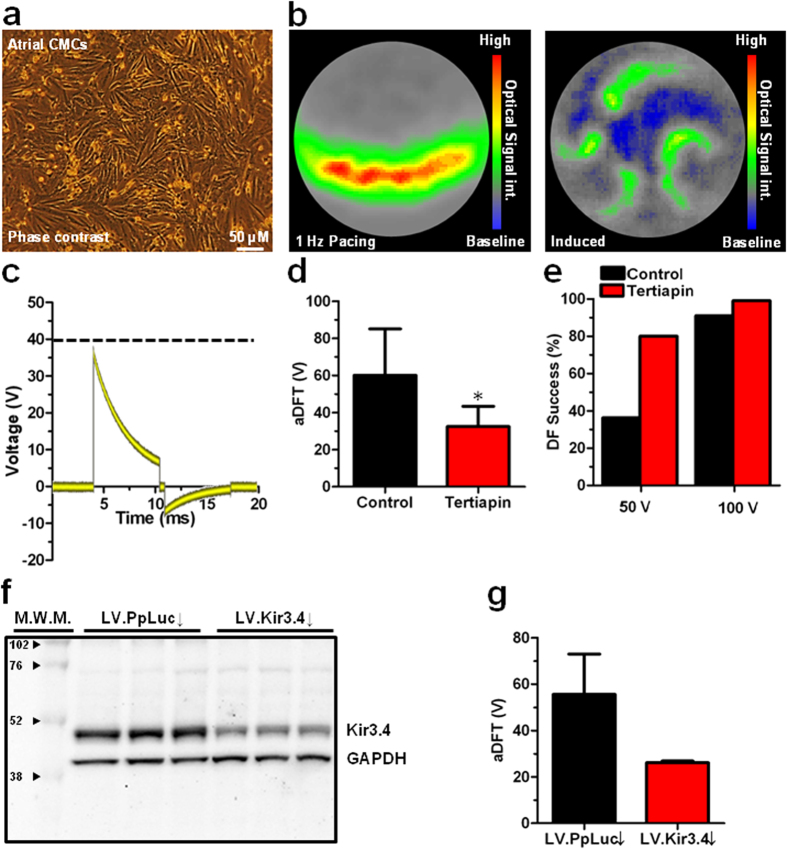
Model characterization and effect of I_K,ACh-c _modulation on aDFT. (**a**) Typical phase-contrast image of a confluent aCMC monolayer. (**b**) Snapshot of the spatial- and high-pass-filtered optical signal in an aCMC monolayer during 1-Hz pacing (left panel) showing a normal uniformly propagated convex wavefront, and after burst pacing displaying complex spiral wave reentry as a 2D model of AF (right panel). (**c**) Typical example of an oscilloscope output during a 40-V biphasic truncated exponential shock. Dashed line indicates the peak voltage of the first phase used in further analyses. Quantification of (**d**) percentage of successful defibrillation (DF) at 50 and 100 V and (**e**) atrial DF threshold (aDFT) in control and tertiapin-treated cultures. (**f**) Western blot of Kir3.4 expression in LV.Kir3.4↓-transduced aCMC cultures and in LV-PpLuc↓-transduced control aCMC cultures using glyceraldehyde 3-phosphate dehydrogenase as loading control. M.W.M: molecular weight marker (numbers are in kDa). (**g**) Quantification of aDFT in aCMC cultures transduced with LV.PpLuc↓ or LV-Kir3.4↓. *p < 0.05 vs control; ^#^p < 0.05 vs LV.PpLuc↓.

To test the hypothesis that I_K,ACh-c_ affects the atrial defibrillation success rate or the aDFT, fibrillating atrial cardiomyocyte (aCMC) cultures were treated with tertiapin (a blocker of I_K,ACh-c_) before being subjected to electric shocks of 25–100 V in order to determine the DFT. Tertiapin treatment notably increased the success rate of defibrillation compared to untreated controls (from 36.3 to 80.0% at 50 V and from 91.0 to 100.0% at 100 V, Fig. 1d). The increase in success rate was accompanied by a significant decrease in DFT from 60.0 ± 25.1 V in control aCMC cultures (n = 33) to 35.5 ± 10.9 V in tertiapin-treated aCMC cultures (n = 10; p < 0.05) (Fig. 1e).

### I_K,ACh-c_ blockade decreases aDFT by increasing wavelength

To elucidate the mechanisms by which I_K,ACh-c_ blockade decreased the DFT, we first investigated the effects of tertiapin on several electrophysiological parameters (*i.e.* conduction velocity [CV], action potential [AP] duration at 80% repolarization [APD_80_], wavelength, reentry cycle length and complexity). Tertiapin (n = 10) significantly increased APD_80_ in aCMCs during reentry (54.0 ± 20.7 vs 25.8 ± 4.6 ms in control cultures [n = 33]) (Supplemental Fig. 2A,B), in the absence of acetylcholine, confirming the presence of constitutively active I_K,ACh_ in the aCMC cultures^18^. The CV during reentry was not significantly affected (9.5 ± 2.8 vs 11.7 ± 2.9 cm/s in control cultures) (Supplemental Fig. 2A,C). Hence, the wavelength (determined by the product of APD and CV) was significantly increased by tertiapin treatment (0.48 ± 0.15 vs 0.30 ± 0.10 cm in control cultures) (Supplemental Fig. 2A,D). Through this increase in wavelength, I_K,ACh-c_ blockade both caused a significant increase in reentry cycle length (106.2 ± 28.5 vs 61.9 ± 18.1 ms in control cultures) (Supplemental Fig. 2A,E) and decrease in arrhythmia complexity (2.5 ± 3.5 vs 5.06 ± 5.06 PSs in control cultures) (Supplemental Fig. 2A,F).

Subsequently, DFT was plotted as a function of CV, APD_80_, wavelength, reentry cycle length or complexity. As shown in Supplemental Fig. 3A, CV did not show any apparent relationship with DFT (R^2^ = 0.12). Furthermore, both reentry cycle length and APD_80_ displayed a weak inverse (hyperbolic-like) relationship with DFT (R^2^ = 0.24 and 0.34, respectively) (Supplemental Fig. 3B,C). DFT seemed to increase concordantly with the complexity of the arrhythmia, although this correlation was not very strong (R^2^ = 0.51, Supplemental Fig. 3D). In contrast, a more apparent relationship was found between wavelength and DFT, which showed an increase in DFT with decreasing wavelengths (R^2^ = 0.65, Supplemental Fig. 3E). Together, these results suggest that a blockade of I_K,ACh-c_ decreases DFT through the resulting increase of the wavelength during reentrant tachyarrhythmias.

### Effect of *Kcnj5* knockdown on electrophysiological parameters and aDFT

To exclude the possibility that the DFT-lowering effect of tertiapin was caused by another mode of action than by its ability to block I_K,ACh-c_, *Kcnj5* expression in aCMC cultures was specifically downregulated by RNAi using LV.Kir3.4↓. After transduction with LV.Kir3.4↓, Kir3.4 levels decreased to 36.5 ± 3.6% of those in LV.PpLuc↓-transduced control cultures), as judged by Western blot analyses (Fig. 1f). Optical mapping of these cultures during reentry yielded similar results as obtained following tertiapin treatment, being a significant increase in APD_80_ (Supplemental Fig. 4A,B), a non-significant decrease in CV (Supplemental Fig. 4A,C) and a significant increase in wavelength (Supplemental Fig. 4A,D) and reentry cycle length (Supplemental Fig. 4A,E), while arrhythmia complexity significantly decreased (Supplemental Fig. 4A,F). Importantly, also the DFT was significantly decreased by *Kcnj5* knockdown (25.0 ± 3.5 [n = 14] vs 50.7 ± 19.4 V in LV.PpLuc↓-transduced cultures [n = 13]) (Fig. 1g). Trends in the relationship between aDFT and CV, APD_80_, wavelength, reentry cycle length or complexity were similar to those found in the tertiapin experiments described in Supplemental Fig. 3 (data not shown).

### Successful defibrillation depends on forced PS removal

To investigate how an increase in wavelength by I_K,ACh-c_ blockade or *Kcnj5* knockdown decreases aDFT, mechanisms of successful and failed defibrillation were studied.

**Figure 2:**
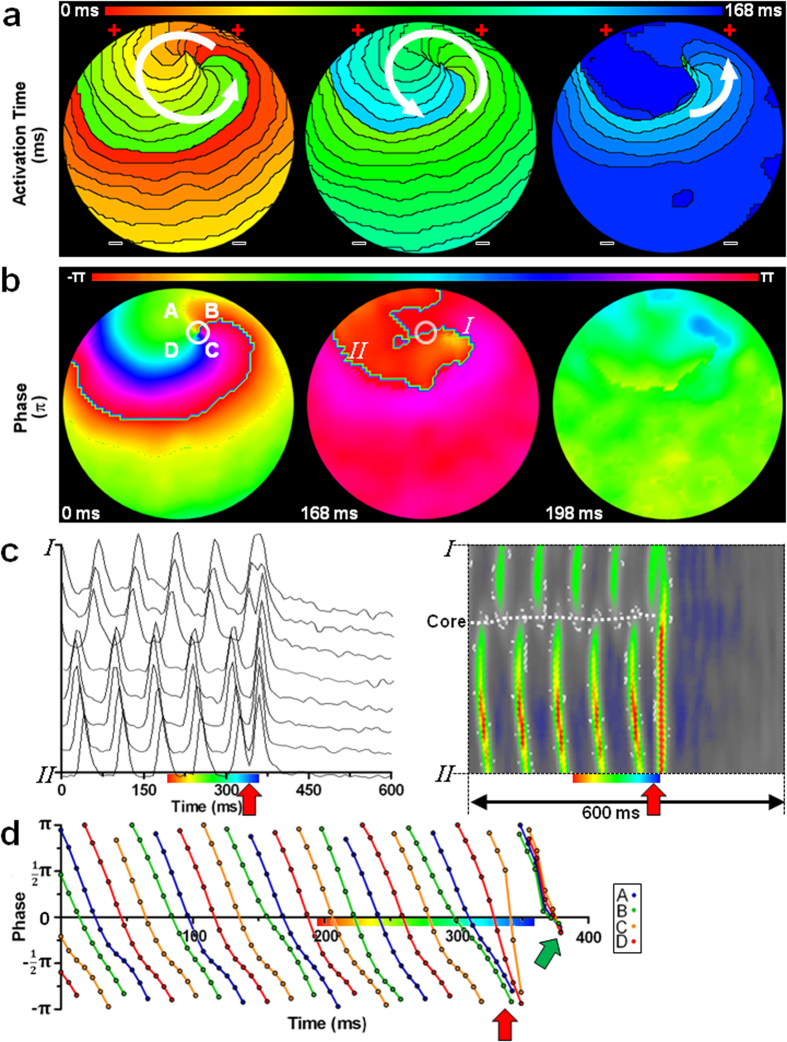
Successful defibrillation. (**a,b**) Typical activation (**a**) and phase (**b**) map sequence of an atrial culture exhibiting a single-rotor tachyarrhythmia prior to (left), during (middle) and after (right) exposure to an electric shock above the DFT. To simplify interpretation of the results, an example of limited complexity was chosen. The white arrows and circles indicate the direction of AP propagation and the location of the PS, respectively. +: side of first-phase cathode. –: side of first-phase anode. (**c**) Line analysis of “optical APs” (left) and filtered optical signals (right) (between points I and II in subfigure **b**) showing synchronization of depolarization after electrical shock application (indicated by the red arrow). Dashed white line indicates the position of the functional core of reentry. Color bar below the X-axis corresponds to that in subfigure **a**. (**d**) Plot of the phase at four points equally spaced around the PS (points A, B, C and D in subfigure **b**) prior to, during and after application of an electrical shock (indicated by the red arrow), showing completely asynchronous phase progression before but full synchronization of all phases (green arrow) after delivery of the electrical shock.

During reentry, a continuous spatial progression of activation and phase around a PS (the point surrounded by a complete cycle of phase [2π])^21^ (Fig. 2a,b, left pictures) was observed, perpetuating both the PS and its consequent reentrant tachyarrhythmia. Following exposure of such a culture to a high-energy electric shock, this continuous spatial progression of activation was disturbed, through simultaneous depolarization of large areas of the culture (Fig. 2a, right picture; Fig. 2c, red arrow). As a consequence, the electrical shock forced all cells to the same phase of the AP (Fig. 2b, middle picture; Fig. 2d, red and green arrow), breaking the 2π phase convergence necessary for PS maintenance. Hence, after synchronous repolarization (Fig. 2b, right picture; Fig. 2c) reentry was fully terminated (Supplemental Movie 1).

**Figure 3:**
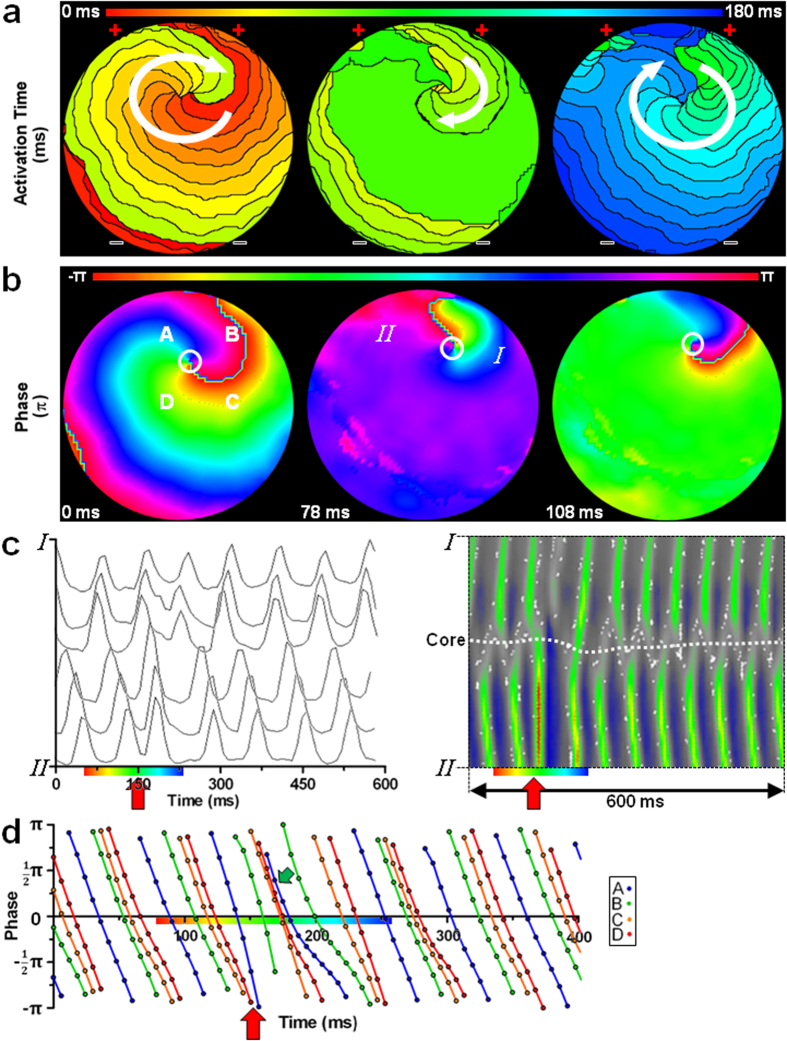
Failed defibrillation caused by incomplete PS removal. (**a,b**) Typical activation (**a**) and phase (**b**) map sequence of an atrial culture exhibiting a single rotor tachyarrhythmia prior to (left), during (middle) and after (right) exposure to an electrical shock below the DFT. To simplify interpretation of the results, an example of limited complexity was chosen. (**c**) Line analysis of “optical APs” and filtered optical signals (between points I and II in subfigure **b**) showing synchronization of depolarization during application of an electrical shock (indicated by the red arrow). (**d**) Plot of the phase at four points equally spaced around the PS (points A, B, C and D in subfigure **b**) prior to, during and after application of an electrical shock (indicated by the red arrow), showing asynchronous phase progression before and incomplete synchronization of all phases (green arrow), mainly at point B, after delivery of the electrical shock resulting in continued asynchronous phase progression. Symbols are equal to those in Fig. 2.

Concordantly, following exposure of a culture to a shock below the DFT, termination of the arrhythmia failed as a consequence of incomplete synchronization of phases around a PS preventing its definitive removal. In these cases, the electrical shock still elicited simultaneous activation of large areas of the cultures (Fig. 3a,c), perturbing the continuous spatial (Fig. 3a,b) and temporal (Fig. 3d) progression of activation and phase around the PS. However, tracking of the spatial and temporal phase progression showed incomplete synchronization of phase (point B in Fig. 3b,d) after the low-energy shock, causing PS persistence and failure of defibrillation (Supplemental Movie 2).

### Failed defibrillation caused by post-shock formation of PSs

**Figure 4:**
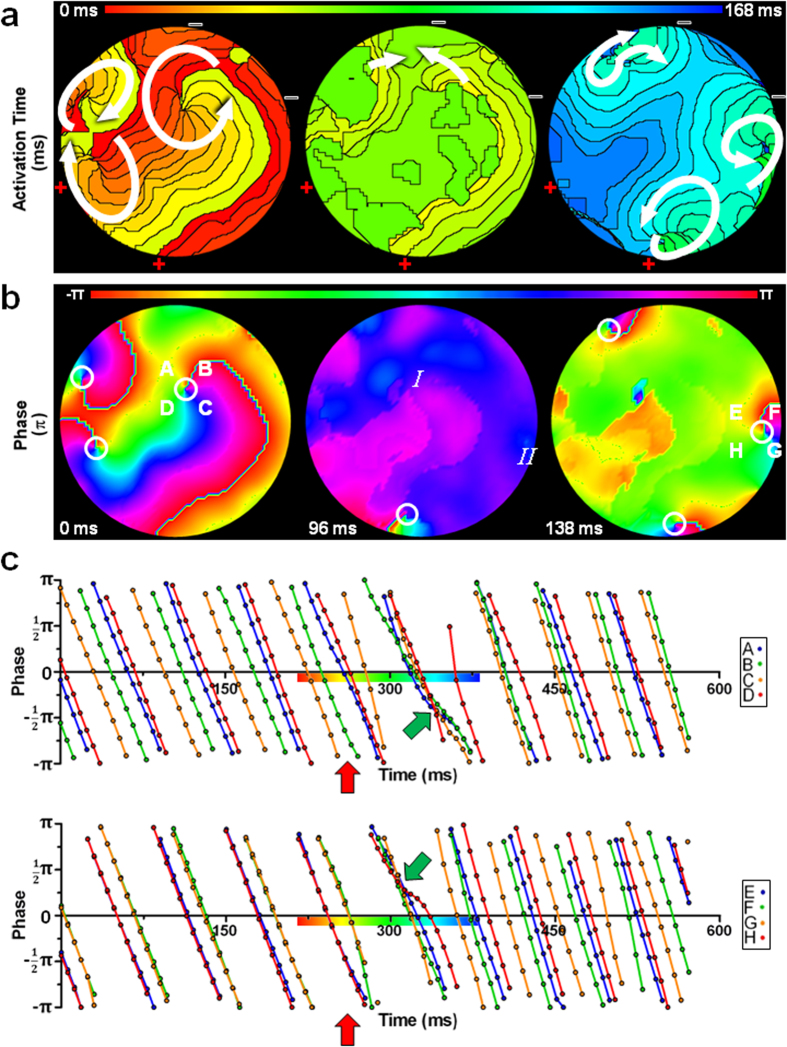
Failed defibrillation caused by re-initiation. (**a,b**) Typical activation (**a**) and phase (**b**) map sequence of an atrial culture prior to (left), during (middle) and after (right) exposure to an electrical shock below DFT. (**c**) Plot of the phase at four points (A, B, C and D in subfigure **b**) equally spaced around a PS that is eliminated by the electrical shock and at four points (E, F, G and H in subfigure **b**) around a PS that emerges after electrical shock application (indicated by the red arrow). Points A, B, C and D showed asynchronous phase progression before and complete synchronization of all phases after delivery of the electrical shock (green arrow), followed by phase progression synchronized at points A and D and B and C due to AP propagation from a different PS. Before application of an electrical shock, points E, F, G and H showed phase progression synchronized at points E and H and F and G as a consequence of AP propagation from a nearby PS. Electrical shock delivery resulted in complete synchronization of all phases (green arrow) after which phase progression proceeded asynchronously as a consequence of a PS arising between these points. Symbols are equal to those in Fig. 2.

**Figure 5:**
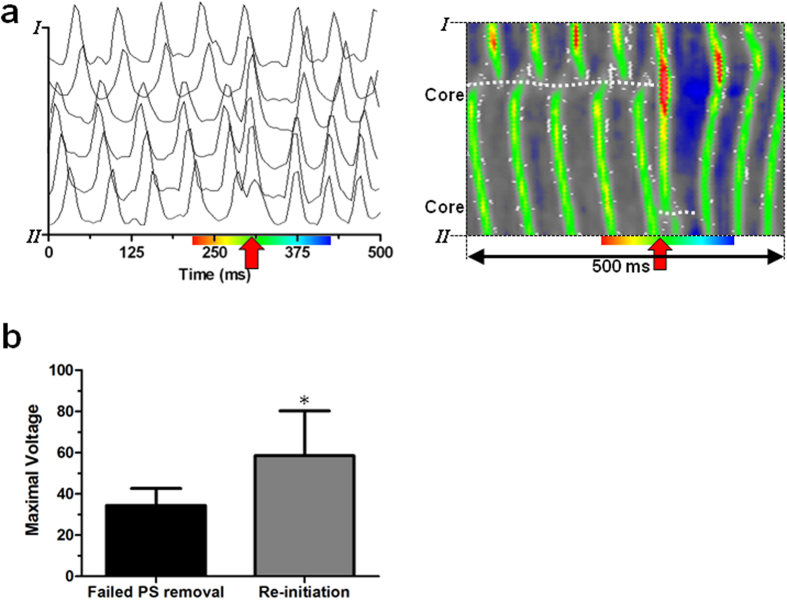
The relative role of re-initiation in failed defibrillation. (**a**) Line analysis of “optical APs” (left) and filtered optical signals (right) showing synchronization of depolarization during application of an electrical shock (indicated by the red arrow). Points I and II correspond to points I and II in Fig. 4b. Dashed white lines indicate the positions of the functional cores of reentry. Color bar below the X-axis corresponds to that in Figure 5a. (**b**) Quantification of the maximal voltage during a 25–100 V incremental shock protocol at which defibrillation failed because of failed phase singularity (PS) removal or post-shock PS formation leading to re-initiation of reentry. *p < 0.05 vs failed synchronization.

Interestingly, application of electrical shocks to aCMC cultures during fibrillation at voltages that eliminated all PSs present prior to the shock did not necessarily lead to successful defibrillation. In such instances, the gradual spatial progression of activation and phase present prior to shock (Fig. 4a,b, left pictures) was again interrupted by simultaneous activation of large areas of the culture, leading to phase synchronization and elimination of the existing PSs (Fig. 4b, middle picture; Fig. 4c,d, green arrows and Fig. 5a). However, if the shock strength was below DFT, new PSs formed in the culture prior to its full repolarization. The newly formed PSs again gave rise to reentrant activation, reestablishing fibrillation in a different activation pattern (re-initiation), causing a failed defibrillation attempt (Fig. 4a,b, right pictures; Fig. 4c,d; Supplemental Movie 3).

Importantly, failure of defibrillation as a consequence of re-initiation was found to occur at significantly higher voltages (*e.g.* closer to the DFT) than failure of defibrillation caused by failed PS removal (58.6 ± 21.6 vs 34.5 ± 8.2 V) (Fig. 5b). This indicates that of the mechanisms responsible for failed defibrillation, re-initiation is more important in setting the DFT. Together these results suggest that an increase in wavelength by I_K,ACh-c_ blockade might decrease DFT by suppressing the occurrence of post-shock PS formation.

### Mechanism of post-shock re-initiation

To elucidate the mechanism underlying post-shock formation of PSs ,we first subjected control atrial cultures (n = 10) to 25–100 V shocks without prior induction of reentry or electrical stimulation. In none of these cultures post-shock emergence of PSs was observed (Supplemental Fig. 5A,B), ruling out the shock protocol or configuration as a cause of post-shock re-initiation of reentry.

**Figure 6:**
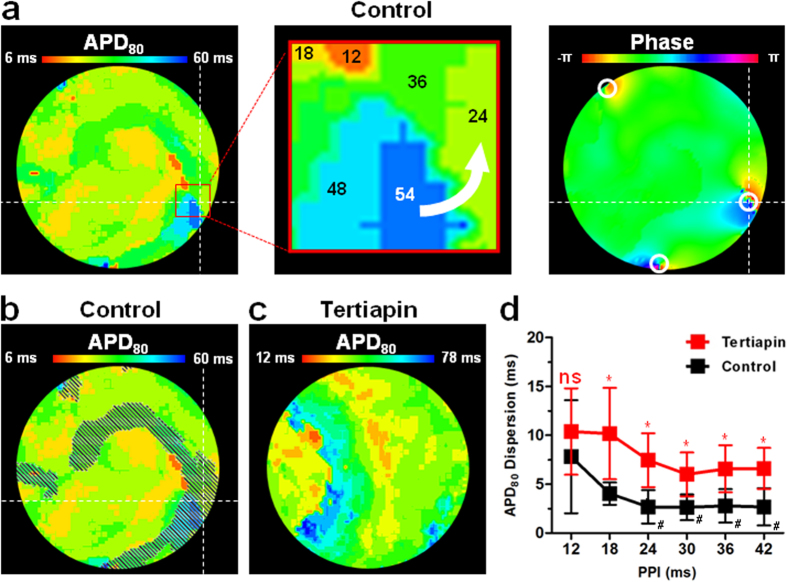
Repolarization heterogeneities and failed defibrillation. (**a**) Map of AP duration until 80% repolarization (APD_80_) (left panel) of the shock-induced AP and phase map (right panel) of a control culture directly after electrical shock delivery, showing that the locations of the steepest APD/repolarization gradients co-localize with the areas of PS formation directly after the shock. The magnified area (red square) of the repolarization map (upper right panel) indicates the APDs and the direction of AP propagation going from the area of latest repolarization towards the area of earliest repolarization (white arrow). (**b**) Overlay of the areas with 6- to12-ms PPIs (grey hatching) and the APD_80_ map, showing that areas of steep APD gradients fall in the areas with the shortest PPIs. (**c**) Repolarization map (left panel) of the shock-induced AP in a tertiapin-treated culture. (**d**) Quantification of APD_80_ dispersion of APs induced by 30-V electrical shocks in tertiapin-treated and control cultures at PPIs of 12 to 42 ms.

**Figure 7:**
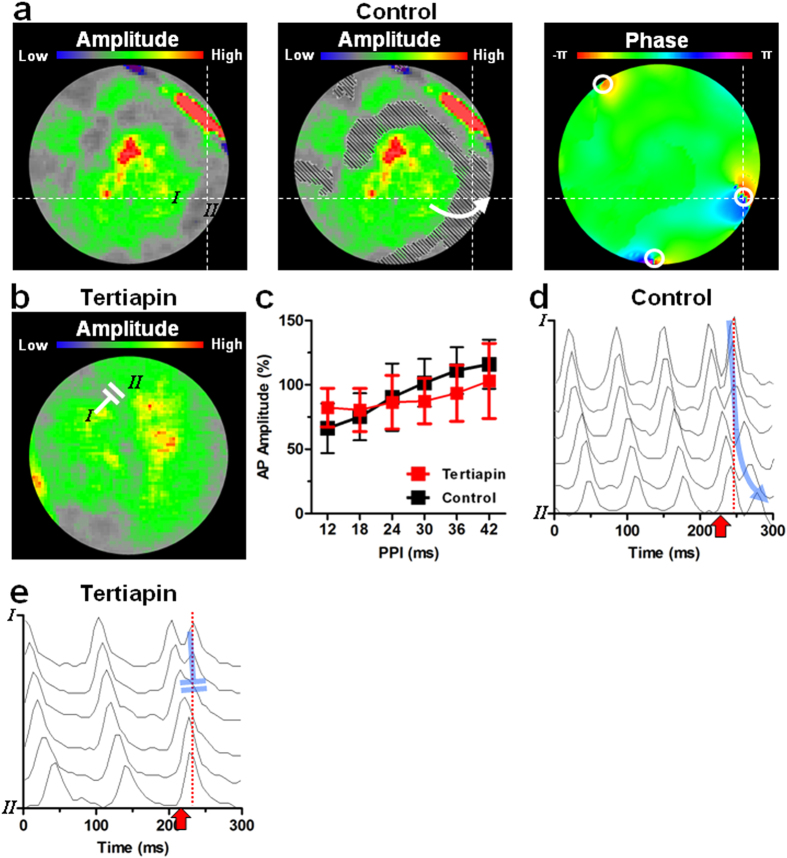
Depolarization heterogeneities and failed defibrillation. (**a**) Map of the amplitude of the shock-induced AP without (left panel) or with an overlay of the areas with 6- to 12-ms PPIs (grey hatching; middle panel) and the phase map of a control culture directly after delivery of a 30-V electrical shock, showing co-localization of short PPIs, low shock-induced AP amplitudes and sites of post-shock PS emergence. (**b**) Map of the amplitude of the shock-induced AP of a tertiapin-treated culture. (**c**) Quantification of the shock-induced AP amplitude in control and tertiapin-treated cultures at PPIs between 12 and 42 ms. (**d**) Filtered optical signals between points I and II depicted in subfigure (**a**), showing impaired shock-induced depolarization near point II, leading to propagation of the shock-induced AP wavefront towards point II (direction indicated by blue arrow and white arrow in A). (**e**) Filtered optical signals between points I and II depicted in subfigure **(b**), showing block of conduction (indicated by double blue lines and double white lines in A) of the shock-induced AP wavefront on the repolarizing waveback of the pre-shock activation between areas of large and impaired shock-induced depolarization.

Next, we studied the repolarization of fibrillating control or tertiapin-treated cultures after applying shocks with a magnitude below the DFT established for control conditions (*i.e.* 30 V). This showed that the position of newly formed PSs co-localized with the steepest gradients in APD after the shock, with the post-shock reentrant wavefront moving from the area of latest towards to area of earliest repolarization (Fig. 6a). Dispersion in duration of the shock-induced AP was found to be significantly higher at the shortest pre-shock peak-to-post-shock peak intervals (PPIs) (Fig. 6b,d). I_K,ACh-c_ blockade by tertiapin, did not prevent dispersion in repolarization of such shock-induced APs (Fig. 6c,d). In fact, post-shock APD dispersion in the tertiapin-treated cultures was significantly higher at diastolic intervals from 18–42 ms (Fig. 7d). Still, the maximal post-shock APD dispersion (*i.e.* at the shortest diastolic interval) did not significantly differ between control and tertiapin-treated cultures. Therefore, to actively assess the contribution of APD dispersion to re-initiation, we created permanent repolarization heterogeneities in aCMC cultures, by transducing the left half of the cultures with LV.Kir3.4↓ (or LV.PpLuc↓ as control), while leaving the right half of the cultures untransduced as described in the Supplemental Material online (Supplemental Figs 6, 7). Interestingly, such spatially-defined downregulation of Kir3.4 significantly lowered the defibrillation threshold (29.2 ± 4.6 vs 41.1 ± 13.5 V in heterogeneously PpLuc↓-transduced control cultures) (Supplemental Fig. 7F). Moreover, analysis of the area of first activation after applying shocks below the DFT showed that these sites were only found in the untransduced part (area I) of LV.Kir3.4↓-transduced aCMC cultures (n = 10) (Supplemental Fig. 7G). In comparison, in LV.PpLuc↓-treated cultures, no clear preference in re-initiation site was found, *i.e.* 40% of the first post-shock APs originated in the untransduced area (area I) vs 10% and 20% in the border area (area II) and transduced area (area III), respectively; n = 10) (Supplemental Fig. 7G). Together these results suggest that re-initiation and prevention of re-initiation by I_K,ACh-c_ inhibition are not driven by repolarization heterogeneities in our model.

Subsequently, to actively assess the role of shock-induced depolarization in re-initiation, we quantified the effect of the PPI on the amplitude of the shock-induced APs at 30 V (*i.e.* below the average DFT) and at 90 V (*i.e.* above the average DFT) during fibrillation. Shock-induced AP amplitudes were significantly higher at 90 V than at 30 V (Supplemental Fig. 8A). The shock-induced AP amplitude diminished significantly with decreasing PPIs at both voltages (Supplemental Fig. 8A). Interestingly, 90-V shocks flattened the curve of the relationship between PPI and amplitude in comparison to 30-V shocks (n = 6 cultures per group), attributable to its increased effect on relative refractory tissue at short PPIs. Concordantly, in the absence of reentry or prior to electrical stimulation there was no difference in the AP amplitude induced by a 30 or 90 V shock (Supplemental Fig. 8B, n = 6 cultures per group), while the functional core of the reentrant spiral waves did not affect shock-induced depolarization (as judged by the effect of distance to the PS on the local shock-induced AP amplitude, Supplemental Fig. 8C, n = 6 cultures per group).

Importantly, the areas of PS emergence after the electrical shock also co-localized with the (borders of) areas of lowest shock-induced AP amplitude (*i.e.* at the areas with the shortest PPIs) (Fig. 7a), with conduction of the shock-induced AP wavefront directed from highly depolarized towards the incompletely depolarized regions (Fig. 7a,d). Tertiapin did not significantly affect the shock-induced AP amplitude (Fig. 7c). However, similar to high voltage shocks, tertiapin treatment showed a trend to flatten the curve of the relationship between PPI and shock-induced AP amplitude (Fig. 7c). Moreover, at areas of heterogeneous shock-induced depolarization in tertiapin-treated cultures, the shock-induced AP wavefront blocked on the refractory tail of the pre-shock activation, as a consequence of the tertiapin-dependent increase in wavelength (Fig. 7e).

Taken together, these results suggest that failed defibrillation through re-initiation is caused by heterogeneous depolarization as a consequence of impaired tissue depolarization at short PPIs. This causes steep post-shock membrane potential gradients, which allow unidirectional propagation of the shock-induced AP in these critical areas. I_K,ACh-c_ blockade blunts the heterogeneity in depolarization induced by low-voltage shocks and inhibits the unidirectional propagation of shock-induced APs by increasing wavelength, which prevents re-initiation and lowers DFT.

### Effect of I_K,ACh-c_ blockade in whole-heart electrical cardioversion

**Figure 8:**
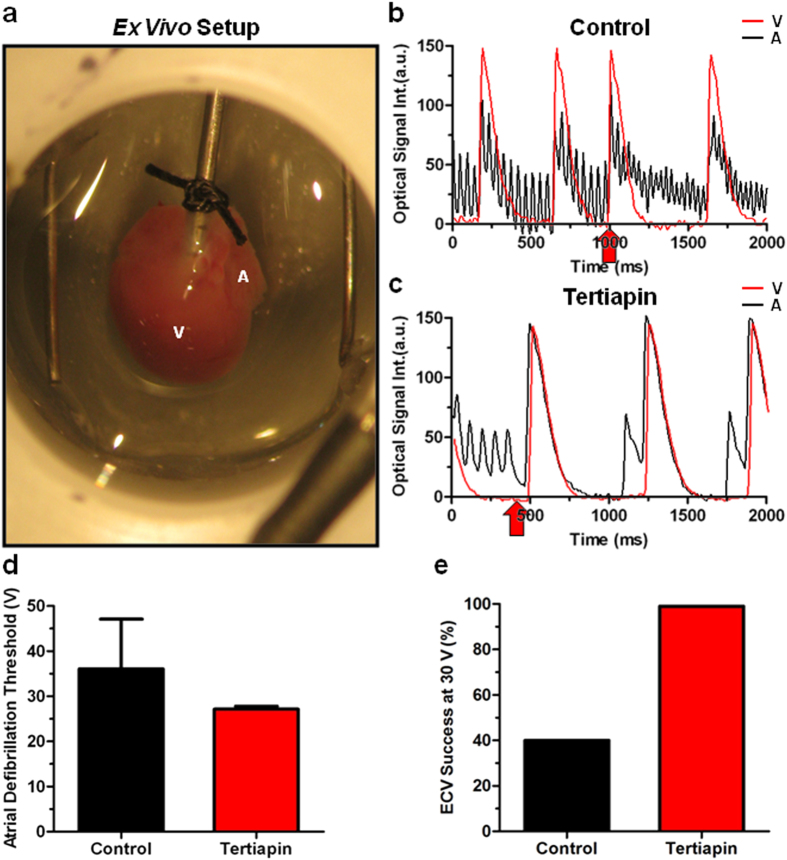
Ex vivo confirmation of the role of I_K,ACh-c_ in determining aDFT. (**a**) Example of a neonatal rat heart in the *ex vivo* mapping setup, prior to commencing the AF induction protocol. Typical ventricular (V) and atrial (**b**) single-pixel recordings of (**b**) a control (n = 6, 16.7% non-inducible) and (**c**) a tertiapin-treated (n = 7, 57.1% non-inducible) heart. Red arrows indicate the moment of electrical shock application. Notice the increase in atrial activation frequency following electrical shock application in the control heart. Quantification of (**d**) atrial defibrillation threshold (aDFT) and (**e**) electrical cardioversion (ECV) success rate following delivery of a 30-V electrical shock in control and tertiapin-treated hearts. a.u.: arbitrary units.

As many of the events investigated in the present study may occur subepicardially in the whole heart, our 2D AF model allowed us to obtain unique insights into the mechanisms determining aDFT. However, to confirm the aDFT lowering effect of I_K,ACh-c_ blockade in the more complex and clinically relevant 3D setting, we studied the effect of I_K,ACh-c_ blockade by tertiapin during whole-heart mapping (Fig. 8a). Indeed, using the lowest defibrillation energy (30 V), failed defibrillation was observed in control hearts. Re-initiation of fibrillation was a major mechanism of failed defibrillation, because a notable change in cycle length could be observed after the electrical shock, suggesting that a different reentrant pathway maintained subsequent fibrillation (Fig. 8b). Following tertiapin treatment, hearts displayed a strong increase in atrial APD and cycle length after AF induction (Fig. 8c), and showed termination of AF by ECV at the lowest voltage (30 V). As expected from the *in vitro* experiments, tertiapin evidently decreased the ECV threshold (36.1 ± 11.3 vs 27.2 ± 0.66 V in untreated control hearts) (Fig. 8d), while the success rate of ECV at 30 V shocks was increased by 60% (100% vs 40% in control hearts) (Fig. 8e). Together, these results support the notion that I_K,ACh-c_ increases ECV threshold and decreases success rate of atrial defibrillation in the whole heart.

## Discussion

Key findings of this study are: (1) Pharmacological blockade of I_K,ACh-c_ in neonatal rat aCMC cultures or intact atria exhibiting persistent spiral waves of electrical activity, results in a significant increase in the wavelength of reentry and decrease in the energy required for electrical defibrillation (*i.e.* a decreases in aDFT). Similar results were obtained after RNAi-mediated downregulation of one of the molecular correlates of I_K,ACh-c,_ Kir 3.4. (2) I_K,ACh-c_ inhibition lowers aDFT by preventing post-shock arrhythmia re-initiation following defibrillation attempts with low voltage shocks. (3) Mechanistically, blockade of I_K,ACh-c_ moderates the spatial shock-induced differences in depolarization and prevents propagation of the shock-induced AP wavefront, through the increase in wavelength. (4) Finally, I_K,ACh-c_ may serve as a target for lowering the energy requirements for ECV and increasing its effectiveness, without altering ventricular electrophysiology, thereby limiting the harmfulness of (pharmacologically assisted) ECV.

AF is known to be a progressive disease; patients with paroxysmal AF therefore often develop persistent or even permanent AF^2^,^3^. The underlying principle of AF begetting AF, is one of the reasons for applying cardioversion in AF patients. Moreover, with disease progression, increasing amounts of energy may be required for successful ECV and patients with permanent AF may ultimately become refractory to cardioversion^9^,^10^,^22^. As the success rate of ECV already decreases after 24 h of AF^22^, this decrease is likely (at least partially) attributable to electrical remodeling (as opposed to its structural counterpart). In this study, we have elaborated on the effect of electrical remodeling on ECV success rate. We show that I_K,ACh-c,_ as a constituent of this remodeling, is a sufficient cause for an increase in aDFT and a decrease in ECV success rate. Since the molecular components of I_K,ACh-c_ are absent in the ventricles, blockade of I_K,ACh-c_ might provide an atrium-specific target to facilitate successful ECV of AF without increasing the risk of ventricular pro-arrhythmia, thereby adding to the significance of our results.

According to present theories, defibrillation will fail if the portion of the myocardium that is excited or rendered refractory by the electrical shock is too small to effectively cause termination of all reentrant conduction^23^,^24^. Indeed, the present study confirms that exciting a critical portion of the myocardium is essential for successful defibrillation. If the shock strength is too low to meet this requirement, the consequent incomplete synchronization of the gradual progression of phase around the PS will lead to its continued existence and thereby to ineffective defibrillation. However, we show that close to the DFT, failure of electrical shocks to defibrillate may occur even if their strength is enough to cause elimination of all pre-existing PSs^25^,^26^,^27^. Here, post-shock formation of PSs is the underlying cause of defibrillation failure. Hence, extinguishing the wavefronts that perpetuate fibrillation through synchronous depolarization is necessary but not sufficient for successful defibrillation.

While other studies, mostly focusing on ventricular fibrillation, have postulated re-initiation of fibrillation as an important mechanism of defibrillation failure of electrical shocks near the DFT, controversy remains regarding the origin of post-shock re-initiation. The presence of an isoelectric window after a failed defibrillation shock (*i.e.* the temporal gap between the application of a shock and the observation of first epicardial activation) was the first clue that led to the introduction of the theories postulating re-initiation of reentry to underlie defibrillation failure^27^. Because of this gap it has been hypothesized that the re-initiation of (ventricular) fibrillation could be caused by triggered activity resulting from early (EADs) or delayed afterdepolarizations (DADs)^28^. In the present study post-shock EADs and DADs were not observed. Moreover, APD prolongation was shown to prevent post-shock re-initiation of reentrant activity. If in our case formation of the first PS after the electrical shock would depend on phase-2 EADs, APD prolongation would increase the chance for re-initiation as well as the DFT. However, APD prolongation does decrease the susceptibility for post shock late phase-3 EADs and DADs^27^. Still, in contrast to our findings, these phenomena are associated with long isoelectric windows or immediate recurrence of AF (IRAF) after primary ECV success^29^. Our results therefore suggest that afterdepolarizations are an unlikely cause of defibrillation failure in AF. Given the rapid reappearance of fibrillatory electrical activity after unsuccessful ECV (Figs 5 and 8), the isoelectric window in whole hearts could be attributable to intramural reentry occurring before activation becomes measurable at the epicardium.

In theory, the origin of re-initiation could also be the electrical shock itself. Several studies showed that when exposed to external field stimulation, areas of cardiac tissue can be either excited (*i.e.* the virtual cathode) or de-excited (*i.e*. the virtual anode)^30^,^31^. The latter causes AP shortening, which restores excitability in the de-excited area^26^,^32^. As a consequence of electrotonic interaction between the depolarized and the hyperpolarized area (*i.e*. break excitation)^33^. new wavefronts can be formed and propagated in the direction of the excitable de-excited area after the shock, potentially creating new reentrant circuits that cause shock failure^26^,^34^.

In the present study, de-excitation was far less apparent (although small areas of APD shortening were observed, analogous to virtual anodes). As tissue heterogeneity may be a determinant of the occurrence of virtual electrode polarization^35^,^36^, this difference may, in part, be attributed to the homogeneous nature of our cell culture model. More importantly, virtual electrode polarization may be prevented by the use of a biphasic shock, as utilized in our study. The second phase of a biphasic shock is thought to quickly re-excite the de-excited area, while only partially de-exciting the excited area, homogenizing post-shock polarization^26^. As such, the effectiveness of the biphasic shock in preventing virtual electrode formation depends on the amplitude of the second phase relative to that of the first phase. In the present study, the peak voltage of the second phase was 20% of the maximum voltage in the first phase, which has been previously shown to cause effective asymmetric reversal of the polarity in the first phase, thereby preventing virtual electrode formation^26^.

Moreover, according to the virtual electrode hypothesis of defibrillation strong shocks can prevent virtual electrode polarization and re-initiation by enlarging the areas of complete de-excitation. If a wavefront is formed by break excitation, this wavefront will promptly excite the de-excited area as a consequence of its local shock-induced gain in excitability. As this fast excitation does not allow for recovery of the virtual cathode area, new reentrant circuits are prevented^32^. In our model, strong shocks do not increase de-excitation (again as a consequence of its biphasic nature), but increase the amount of excited tissue (that would have been refractory to low shock voltages). As a consequence post-shock polarization is homogenized, which prevents re-initiation. Similarly, tertiapin decreases the steepness in the relationship between the shock-induced AP amplitude and the PPI, which also homogenized post-shock polarization. Simultaneously, tertiapin increases refractoriness in incompletely depolarized areas and hence prevents propagation of wavefronts initiated at depolarized areas, which together decrease DFT. Hence, our paper shows that DFT can be decreased through other means than by increasing de-excitation, in the context of a biphasic shock. Still, mechanisms of re-initiation found in our study are largely analogous to the virtual electrode hypothesis being dependent heterogeneous polarization.

Finally, the critical point hypothesis of defibrillation states that reentry can initiate at critical junctions between the shock-induced extracellular potential gradient and the refractoriness gradient caused by the last pre-shock activation^37^. The present study confirms that re-initiation of AF depends on local tissue refractoriness. It should be noted, however, that the critical point hypothesis stems from experiments in which a monophasic S2 shock was given during phase 2 or 3 of the AP from a plane perpendicular to that of the S1 shocks, causing potential and refractoriness gradients to cross each other^28^,^37^. Applying biphasic shocks during fibrillation, should partly resolve the potential gradient, and cause the refractoriness to be dispersed. At variance with the critical point hypothesis, in our model formation of critical points (*i.e.* post-shock PSs) occurred in the area of highest refractoriness (*i.e*. the shortest PPI), instead of remote from this area.

The fact that most of the current theories about cardiac fibrillation and defibrillation mechanisms come from the field of ventricular fibrillation and can only partly explain our findings, may also indicate that different mechanisms might be responsible for defibrillation failure in ventricular fibrillation and AF, underlining the novelty of our results.

In this study, we utilized models of AF in which tachyarrhythmic activation after burst pacing relies on reentrant activity. As focal mechanisms may also underlie fast activation during AF^38^, the energy necessary to eliminate AF of focal origin, as well as the effect of diminishing I_K,ACh-c_, on the efficiency with which such an arrhythmia can be terminated may be different from what was found in the present study. Still, it should be noted that reentrant re-initiation at critical PPIs can also occur after eliminating a focal source by electrical shock. Hence, in this case a protective effect of I_K,ACh-c_ might be expected. Further studies will be necessary to explore this possibility.

In addition, ECV in the clinical setting not only fails because of true shock failure but also by IRAF in which, after the electrical shock, one or more sinus beats are followed by re-initiation of AF^39^. Only the true shock failure aspect of ECV failure has been investigated in the present manuscript. Since the mechanisms for true shock failure and IRAF may differ, blockade of I_K,ACh-c_ might not prevent ECV failure due to IRAF. Nonetheless, we and others have shown that I_K,ACh-c_ blockade can also prevent the initiation of AF^18^,^40^.

The use of neonatal rat aCMC monolayers allowed us to systematically study the mechanisms of AF in the context of I_K,ACh-c_ inhibition in a controllable environment. These monolayers are, however, inherently different from intact human atria because of their 2D nature and the electrophysiological differences between human and rat aCMCs. Nonetheless, results obtained in our 2D model were reproduced in intact hearts, confirming its relevance. Due to the minute dimensions of the intact neonatal rat heart, crosstalk between the ventricular and atrial optical signal was observed during optical mapping. Still, presence or absence of AF is easily derived from the obtained atrial signal. As a consequence of this crosstalk, no analyses on post-shock reinitiation mechanisms were performed in the whole heart. Hence, we concur that the results of this study are conceptual with respect to ECV in humans and therefore cannot be readily extrapolated to the clinical setting.

## Conclusions

This is the first study to systematically study the role of the I_K,ACh-c_ in atrial defibrillation. The results indicate that remodeling of this current, as occurs during AF, can contribute to an increase in aDFT and failure of ECV. Hence, I_K,ACh-c_ may serve as an interesting target for lowering the energy requirements for ECV and increasing its effectiveness, without altering ventricular electrophysiology, limiting the harmfulness of (pharmacologically assisted) ECV.

## Methods

A detailed description of materials and methods can be found in the Supplementary Material online.

All animal experiments were approved by the Animal Experiments Committee of the Leiden University Medical Center and conformed to the Guide for the Care and Use of Laboratory Animals as stated by the US National Institutes of Health.

### Preparation of aCMC monolayers

Neonatal rat aCMCs were isolated by collagenase digestion and cultured on 15-mm-diameter fibronectin-coated coverslips as described previously^18^. A cell density of 8 × 10^5^ cells/well in 24-well culture plates was maintained throughout the experiments by restricting proliferation (of non-myocytes) through treatment with Mitomycin-C (Sigma-Aldrich, St. Louis, MO) at day 1 of culture.

### RNAi

Self-inactivating lentiviral vectors encoding enhanced green fluorescent protein (eGFP) and a shRNA specific for rat *Kcnj5* (LV-Kir3.4↓) or for *Photinus pyralis luciferase* (LV-PpLuc↓, negative control vector) were generated as described in detail in the Supplementary Material online. Transduction was performed at day 4 of culture. Knockdown of Kir3.4 after transduction with LV-Kir3.4↓ was confirmed using Western blot.

### Optical mapping

High resolution optical mapping was performed at day 9 of culture using di-4-ANEPPS (Life Technologies) as a voltage-sensitive dye and a MiCAM ULTIMA-L imaging system (SciMedia, Costa Mesa, CA) as described previously^18^. For 1-Hz or burst pacing of confluent aCMC cultures (Fig. 1a), a custom epoxy-coated bipolar electrode was used, resulting in normal convex waves (Fig. 1b, left picture) or reentrant spiral waves (Fig. 1b, right picture), respectively. Defibrillation was performed using a custom electroshock module (Supplemental Fig. 1A), which was coupled to two platinum electrodes fixed in a plastic ring that was readily mountable to the top of a 24-well culture plate. Electrodes were 9-mm in length, placed parallel 11-mm apart, and 2 mm above the surface of the culture (Supplemental Fig. 1B–D). This setup produced biphasic truncated exponential shocks of adjustable voltages (≈25–105 V) with the second-phase peak voltage being 20% of the first-phase peak voltage (Fig. 1C). DFT was defined as the voltage used during the first shock (starting from ≈25 V and progressively increasing with 10-V increments) leading to full elimination of arrhythmic activity.

To assess the effect of I_K,ACh-c_ blockade on defibrillation, tertiapin (100 nM) was pipetted in the medium and dispersed by gentle agitation, directly followed by optical mapping.

Whole heart di-4-ANEPPS-mapping was performed on neonatal rat hearts suspended in a 16-mm diameter tissue bath. Hearts were perfused with oxygenated Tyrode’s solution (comprising [in mM] NaCl 130, CaCl_2_ 1.8, KCl 4.0, MgCl_2_ 1.0, NaH_2_PO_4_ 1.2, NaHCO_3_ 24 and glucose 5.5 at pH 7.4) supplemented with 20 mM 2,3-butanedione monoxime (Sigma-Aldrich) to minimize motion artifacts. The burst pacing, electrical shock protocol and electrode setup (mounted to the tissue bath) used to determine the aDFT were equal to those used in the *in vitro* experiments.

### Statistics

Statistical analyses and construction of corresponding graphs were performed using Graphpad Prism 6.0 (Graphpad Software, San Diego, CA). Comparison between two groups was performed using the non-parametric Mann-Whitney U test (for unpaired measurements) or Wilcoxon signed rank (for paired measurements) test. Kruskall-Wallis testing with Bonferoni *post-hoc* correction was used for multiple groups and comparisons. Data were expressed as mean ± standard deviation (SD) for a number (n) of observations. Differences were considered statistically significant if p < 0.05. Non-linear regression curves were constructed by using a robust exponential two-phase decay curve fit. Accuracy of these curves was expressed as the coefficient of determination (R^2^).

## Additional Information

**How to cite this article**: Bingen, B. O. *et al*. Constitutively Active Acetylcholine-Dependent Potassium Current Increases Atrial Defibrillation Threshold by Favoring Post-Shock Re-Initiation. *Sci. Rep.* **5**, 15187; doi: 10.1038/srep15187 (2015).

## Supplementary Material

Supplementary Information

Supplementary Movie 1

Supplementary Movie 2

Supplementary Movie 3
