# Supplementary material for: Constitutively Active Acetylcholine-Dependent Potassium Current Increases Atrial Defibrillation Threshold by Favoring Post-Shock Re-Initiation
Source: Sci Rep. 2015 Oct 21;5:15187. doi: 10.1038/srep15187 (PMC4613729; doi:10.1038/srep15187)
Supplement: Supplementary Information [file srep15187-s1.doc]

**Constitutively Active Acetylcholine-Dependent Potassium Current Increases Atrial Defibrillation Threshold by Favoring Post-Shock Re-Initiation.**

Role of I_KACh-c_ in Atrial Defibrillation Threshold.

Brian O. Bingen^1^; Saïd F. A. Askar^1^; Zeinab Neshati^1^; Iolanda Feola^1^; Alexander V. Panfilov^2^; Antoine A. F. de Vries^1^; Daniël A. Pijnappels^1*^

Laboratory of Experimental Cardiology, Department of Cardiology^1^, Leiden University Medical Center, Leiden, the Netherlands.

Department of Physics and Astronomy^2^, Ghent University, Ghent, Belgium.

*Address for correspondence:

D.A. Pijnappels, PhD

Laboratory of Experimental Cardiology

Department of Cardiology

Leiden University Medical Center

2300 RC Leiden, the Netherlands

Phone: +31-(0)71-5262020

Fax: +31-(0)71-5266809

E-mail: D.A.Pijnappels@lumc.nl

**Supplemental material**

**Results**

**Optical mapping of cultures with local transduction of LV.PpLuc↓ or LV.Kir3.4↓**

To assess the contribution of structural repolarization heterogeneities to post-shock PS formation, aCMC monolayers were locally transduced with LV.Kir3.4↓ or LV.PpLuc↓ using patterned attachment, prior to inducing fibrillation and attempting defibrillation. Fluorescence microscopy showed that patterned attachment indeed led to locally increased expression of eGFP in the transduced area (Supplemental Figure 6A). Expectedly, optical mapping of aCMC cultures locally transduced with LV.PpLuc↓ showed similar electrophysiological characteristic in the transduced, the border and untransduced area (depicted as I, II and III in Supplemental Figure 6B respectively, Supplemental Figure 7B-E). In contrast, in cultures with local transduction of LV.Kir3.4↓ a notable increase in the APD was observed in the transduced area during reentry. As a consequence (given the presence of a rotor in the untransduced area) wavebreak occurred in the border (Supplemental Figure 7A, PSs in area depicted as II), with APD alternans occurring in this zone with 2:1 conduction to the transduced area. Hence the difference in APD_80_ (24.7±24.0 vs 1.0±1.1 ms), wavelength (0.05±0.04 vs 0.29±0.26 cm) and activation frequency (4.7±5.7 vs 0.6±1.0 Hz) between the transduced and untransduced area, was significantly higher in the LV.Kir3.4↓ group as compared to controls. The difference in conduction velocity did not show any significant differences (1.9±1.9 vs 3.7±3.6 cm/s) (Supplemental Figure 7B-E). These result confirmed the possibility to establish APD/repolarization heterogeneities in aCMC cultures by patterned attachment.

**Methods**

All animal experiments were approved by the Animal Experiments Committee of the Leiden University Medical Center and conformed to the Guide for the Care and Use of Laboratory Animals as stated by the US National Institutes of Health.

**Preparation of aCMC monolayers**

The isolation and culture of neonatal rat aCMCs was performed essentially as described elsewhere.^1^ Two-Day-old Wistar rats were anesthetized using 5% isoflurane inhalation. Anesthesia was considered adequate if pain reflexes were absent. Subsequently, hearts were excised aseptically, remaining large vessels were removed and atria were separated from the ventricles. Next, atria were gently minced and rinsed in solution A (0.02% phenol red, 137 mM NaCl, 5.4 mM KCl, 0.34 mM Na2HPO4, 0.44 mM KH2PO4, 5.6 mM D-glucose and 20 mM HEPES, pH 7.3) to remove remaining blood. Atrial tissue was dissociated using collagenase (200 U/ml in solution A, Worthington Biochemical Corporation, Lakewood, NJ) and DNAse (6U/ml in solution A, Sigma-Aldrich, St. Louis, MO) in a shaking water bath at 37°C for 2×30 minutes, after which cells were spun down and suspended in Ham's F10 medium (ICN Biomedicals, Irvine, CA, USA) with 10% horse serum (HS, Invitrogen, Carlsbad, CA, USA) and 10% fetal bovine serum (FBS, Invitrogen). Cell suspensions were pre-plated on primaria-coated culture dishes (Becton Dickinson, Franklin Lakes, NJ, USA) to allow preferential attachment of non-myocytes. The suspension of non-adherent cells (mainly aCMCs) was run through a 70-µm cell strainer (Beckton Dickinson) to remove incompletely digested tissue and cell aggregrates, and plated on fibronectin-coated glass coverslips (15-mm diameter) in 24-wells cell culture plates. A cell density of 8×10^5^ cells/well was maintained throughout the experiments by restricting proliferation (of non-myocytes) by treatment with Mitomycin-C (10µg/ml, Sigma-Aldrich, St. Louis, MO) at day 1 of culture. Cultures were kept in a humidified incubator at 37 °C and 5% CO_2_ and refreshed daily with culture medium consisting of a 1:1 mixture of DMEM (Invitrogen) and Ham's F10 supplemented with 5% HS, penicillin (100 U/ml, Life Technologies, Bleiswijk, the Netherlands) and streptomycin (100 μg/ml, Life Technologies).

**Western blotting**

Cultures of aCMCs were lysed in radioimmunoprecipitation-assay buffer comprised of 50 mM Tris–HCl (pH 8.0), 150 mM NaCl, 1% Triton X-100, 0.5% sodium deoxycholate, 0.1% sodium dodecyl sulfate. The proteins in the lysate were size fractionated in Bolt 10% Bis-Tris gels (Life Technologies) and blotted on Hybond polyvinylidene difluoride membranes (GE Healthcare, Diegem, Belgium). Membranes were first incubated with antibodies directed against Kir3.4 (Santa Cruz Biotechnology, Dallas, TX) or glyceraldehyde 3-phosphate dehydrogenase (GAPDH; Merck Millipore, Billerica, MA) as a loading control, followed by incubation with corresponding horseradish peroxidase-conjugated secondary antibodies (Santa Cruz Biotechnology) after blocking in in Tris-based saline, 0.1% Tween-20, 5% bovine serum albumin (Sigma-Aldrich). Chemiluminescence was detected by a ChemiDoc XRS imaging system (Bio-Rad Laboratories, Veenendaal, the Netherlands) using SuperSignal West Femto chemiluminescent substrate (Thermo Scientific, Rockford, IL).

**Fluorescence microscopy**

After a double rinsing step with ice-cold PBS, 10 min fixation in 1% formaldehyde in PBS and 5 min permeabilization with 0.1% Triton X-100 in PBS, cells were stained for nuclei by incubating Hoechst 33342 for 5 min on ice. After double rinsing with PBS, coverslips were mounted in Vectashield mounting medium (Vector Laboratories, Burlingame, CA) to minimize photobleaching. Images were captured using a fluorescence microscope equipped with a digital color camera (Nikon Eclipse 80i; Nikon Instruments Europe, Amstelveen, the Netherlands). Dedicated software (NIS Elements [Nikon Instruments Europe] was used to store immunofluorescence signals.

**RNAi**

Knockdown of Kir3.4 expression in aCMC cultures was performed by transduction with self-inactivating lentiviral vectors (LVs) encoding short hairpin (sh) RNAs specific for rat *Kcnj5* (LV-Kir3.5↓). The *Photinus pyralis* *luciferase (PpLuc)*-specific shRNA-coding sequence and the marker gene cassette consisting of the human *phosphoglycerate kinase 1* gene promoter and the puromycin-N-acetyltransferase-coding sequence in the plasmid SHC007 from the Mission shRNA library (Sigma-Aldrich) were replaced by a rat *Kcnj5*-specific shRNA-coding sequence and the human *eukaryotic translation elongation factor 1 alpha 1* gene promoter and the *Aequorea victoria* enhanced green fluorescent protein-coding sequence, respectively, to acquire the shuttleplasmid constructs to produce aforementioned LVs. The shuttle construct for negative control vector (LV-Ppluc↓) was obtained by omitting the substitution of the *Ppluc*-specific shRNA-coding sequence.

LV particles from the resulting SIN-LV shuttle plasmids, pLKO.1-mKcnj5-shRNA.hEEF1a1.eGFP and pLKO.1-PpLuc-shRNA.hEEF1a1.eGFP were produced as described previously.^1^

Transduction of aCMCs was performed at day 4 of culture by pipetting LV suspension in the culture medium and incubating for 18 hours at a multiplicity of infection that resulted in transduction of essentially all aCMCs.

**Patterned attachment**

To study the effect of Kir3.4 heterogeneity in atrial defibrillation, regional differences in Kir3.4 expression were created using patterned attachment of cardiomyocytes transduced with LV-Kir3.4↓ or LV-PpLuc↓ (as a negative control) and untransduced cells. Directly after aCMC isolation, lentiviral vector suspension was pipetted in half of the aCMC suspension. Custom-made UV-treated Plexiglas inserts were used to occupy the exact upper half (down to 0.5mm from the bottom of the culture to prevent damaging of the fibronectin coating and attached cells later on) of the culture during plating of the transduced cells (4×10^5^ cells/well). After allowing the transduced aCMCs to attach to the bottom half of the culture for 4 hours, medium was removed and cells were rinsed 3 times with PBS to remove unattached cells and remaining lentiviral vector particles. The Plexiglas inserts were moved to the bottom half of the dish after which untransduced cells (4×10^5^) were plated on the upper half of the culture. Plexiglass inserts were removed after 4 hours followed by rinsing with PBS and refreshing of the Ham's F10 medium. Mitomycin-C treatment was performed at day 1 of culture to prevent unwanted proliferation of the non-myocyte fraction. Culturing of confluent monolayers with patterned expression of Kir3.4 and controls followed the standard protocol described above. Analyses were performed at day 9 of culture.

**Optical mapping**

Optical mapping of the action potential propagation using di-4-ANEPPS (Life Technologies) as a voltage sensitive dye and its corresponding analyses were performed essentially using methods described previously.^1,2^ Briefly, aCMC monolayers were incubated with DMEM/HamsF12 containing 8µM di-4-ANEPPS for 15 minutes in the humidified CO_2_ incubator at 37ºC. Following incubation, cells were refreshed with DMEM/HamsF12 and optical images were immediately captured with a MiCAM ULTIMA-L high resolution (100x100 pixels, 160µm/pixel) imaging system (SciMedia, Costa Mesa, CA) at 6ms/frame. Conduction velocity, APD to 80% repolarization APD_80_, reentry cycle length and wavelength (defined as the conduction velocity×APD_80_ during reentry) were calculated using BrainVision Analyzer 1304 (BrainVision, Tokyo, Japan). Pre-shock peak-to-peak interval (PPI) was defined as the time between the peaks of the last pre-shock action potential and shock-induced action potential. Activation maps and line analyses were constructed after high-pass filtering. APD maps, PPI maps and single pixel recordings were non-high-pass and spatially filtered in a 3x3 pixel grid. Complexity was defined as the number of phase singularities (PSs) per culture, determined by using the phase space method as described previously.^1,2^ Defibrillation was performed using a custom electroshock module, capable of producing biphasic truncated exponential shocks of adjustable voltages (Supplemental Figure 1A) coupled to two platinum electrodes fixed in a plastic ring that is readily mountable to the top of a 24-well culture plate. Electrodes were 9-mm in length, placed parallel 11-mm apart, 2 mm above the surface of the culture (Supplemental Figure 1B-D). Peak voltage of the first phase was documented and used in further analyses (Figure 1C). Settings of the electroshock module were calibrated using a DLM4000 digital oscilloscope (Yokogawa, Tokyo, Japan). Successful defibrillation was defined elimination of all reentrant conduction without reinitiation of reentrant conduction after termination. Reinitiation of fibrillation was defined as the occurrence of new PSs and consequent reentrant conduction after elimination of the PS giving rise to the activation wavefront in the respective area within 1000 ms after application after the shock.

To assess the effect of I_K,ACh-c_ blockade on defibrillation, tertiapin (100 nM) was pipetted in the medium en dispersed by gentle agitation, directly followed by optical mapping.

**Ex vivo mapping**

Two-Day-old Wistar rats were anesthetized by isoflurane inhalation (5%). After confirmation of adequate anesthesia by absence of pain reflexes, the hearts were excised and submersed in ice cold Tyrode’s solution (comprising [in mM] NaCl 130, CaCl_2_ 1.8, KCl 4.0, MgCl_2_ 1.0, NaH_2_PO_4_ 1.2, NaHCO_3_ 24 and glucose 5.5 at pH 7.4). Subsequently, the aorta was canulated and retrogradely perfused with oxygenated Tyrode’s solution supplemented with 20 mM 2,3-butanedione monoxime (Sigma-Aldrich) to minimize motion artifacts using a modified Langendorff apparatus (AD instruments, Spechbach, Germany). The heart was submersed in a 16-mm diameter tissue bath containing Tyrode’s solution to allow usage of the same electroshock module as in the aCMC monolayer experiments for defibrillation (see Supplemental Figure 1 and Figure 8A). Hearts were stained with 2 µM di-4-anepps by a 5 ml bolus injection. AF was induced by burst pacing at a cycle length of 20-100 ms using a concentric bipolar platinum/iridium electrode (FHC inc. Bowdoin, ME). To assess the effect of I_K,ACh_ blockade on defibrillation, tertiapin (100 nM) was added to the perfusate as well as the tissue bath.

**Statistics**

Statistical analyses and construction of corresponding graphs were performed using Graphpad Prism version 6.0 software (Graphpad Software, San Diego, CA). Comparison between two groups was performed using the Mann-Whitney U test or Wilcoxon signed rank test where appropriate. Kruskall-Wallis testing with Bonferoni *post-hoc* correction was used for multiple groups and comparisons. Data were expressed as mean±standard deviation (SD) for a number (n) of observations. Differences were considered statistically significant at p<0.05. Non-linear regression curves were constructed by using a robust exponential two-phase decay curve fit. Accuracy of these curves was expressed as the coefficient of determination (R^2^).

**Figures and Legends**

**Supplemental Figure 1.**

**
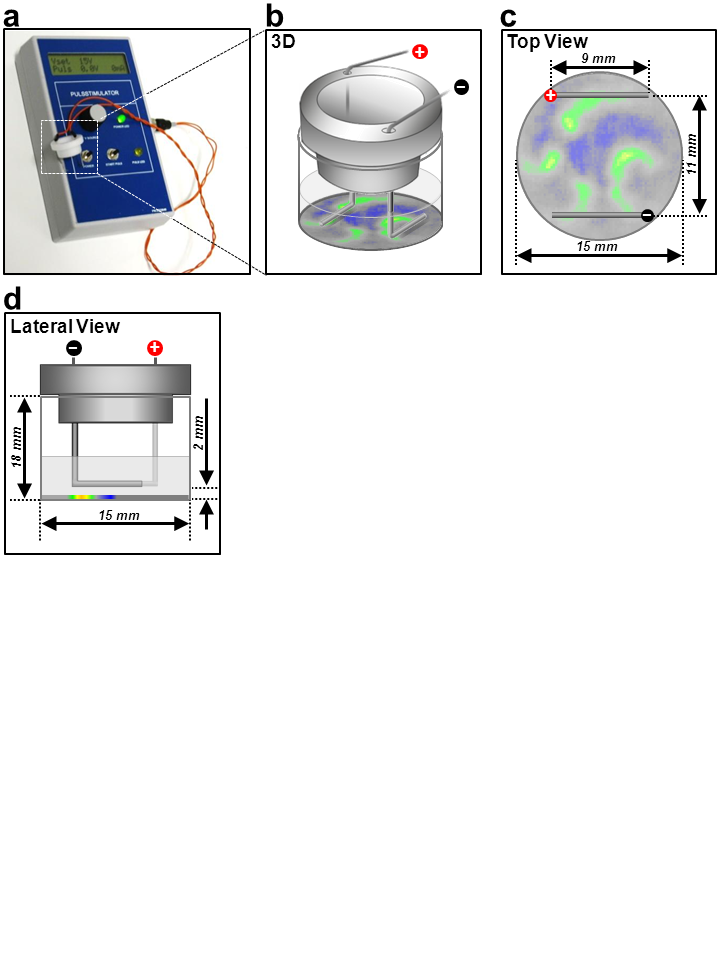
**

*Characterization of experimental setup*. (A) *In vitro* electroshock device consisting of a module capable of generating biphasic truncated exponential shocks at adjustable voltages, coupled to 2 platinum perpendicular electrodes fixed to a plastic rim (dotted square) that is readily mountable to the top of a 24-wells culture plate. (B) Schematic 3D representation, (C) top view (right panel) and (D) lateral view (lower panel) of the used electrodes mounted on a fibrillating atrial culture.

**Supplemental Figure 2.**

**
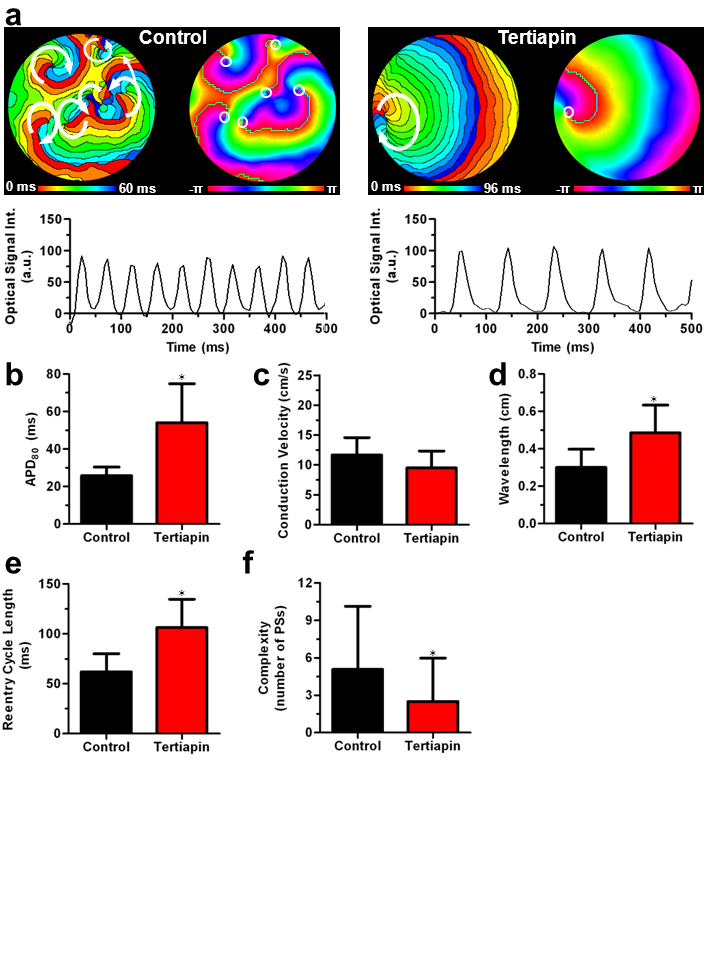
**

*Effect of IK,ACh blockade on electrophysiological characteristics during reentry.* (A) Typical examples of activation maps, phase maps and optical signal during fibrillation induced by burst pacing in control (left), and tertiapin-treated (right) aCMC monolayers. Quantification of (B) APD80, (C) conduction velocity, (D) wavelength, (E) reentry cycle length, and (F) complexity. *:p<0.05 vs control.

**Supplemental Figure 3.**

**
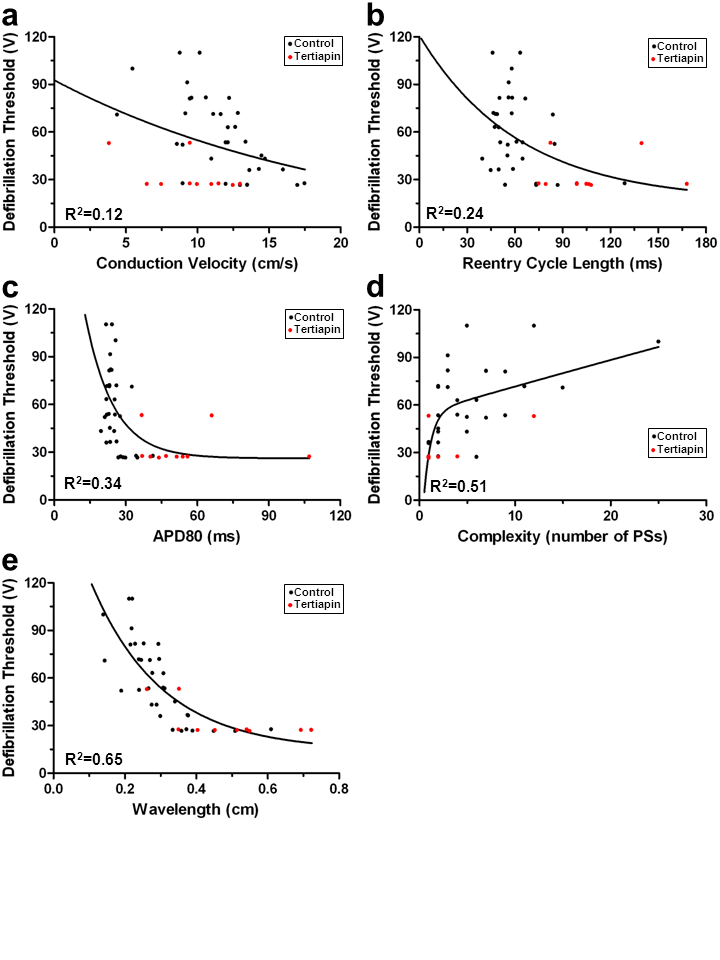
**

*Relationship between electrophysiological parameters and defibrillation threshold.* Plots of the correlation between defibrillation threshold and (A) conduction velocity, (B) reentry cycle length, (C) APD80, (D) complexity and (E) wavelength using control and tertiapin-treated aCMC monolayers.

**Supplemental Figure 4.**

**
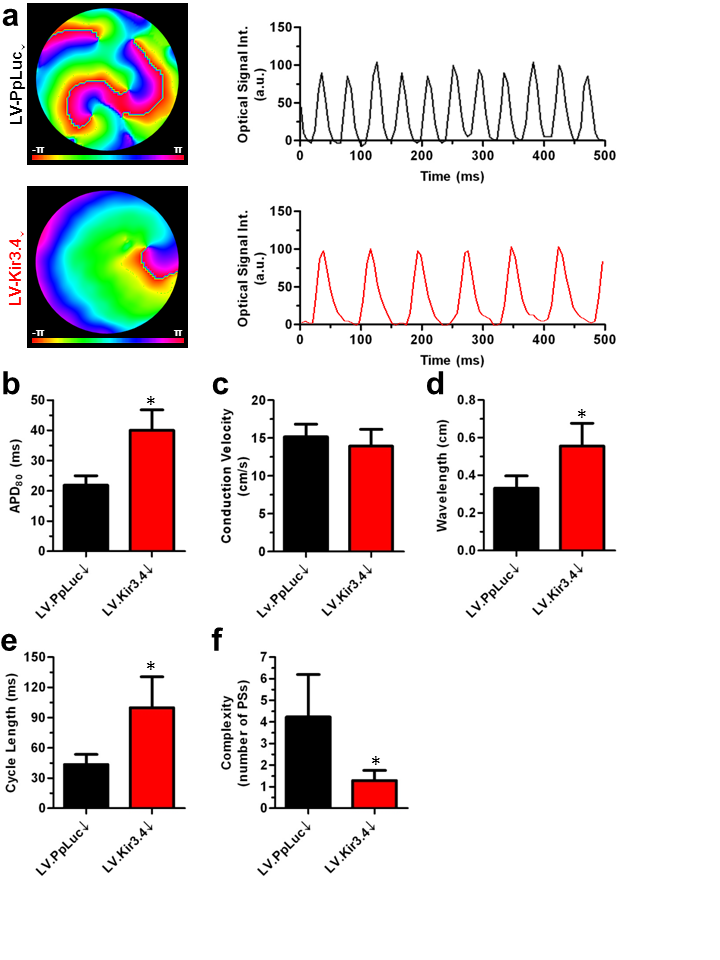
**

*Effect of KCNJ5/2 knockdown on electrophysiological parametersd.*(A) Typical phasemaps and corresponding single pixel recordings in aCMC monolayers transduced with LV.PpLuc↓ (upper paner) or LV-Kir3.1↓ (lower panel). Quantification of (B) APD80, (C) conduction velocity, (D) wavelength, (E) cylcle length, (F) and complexity in aCMC monolayers transduced with LV.PpLuc↓ or LV-Kir3.1↓.

**Supplemental Figure 5.**

**
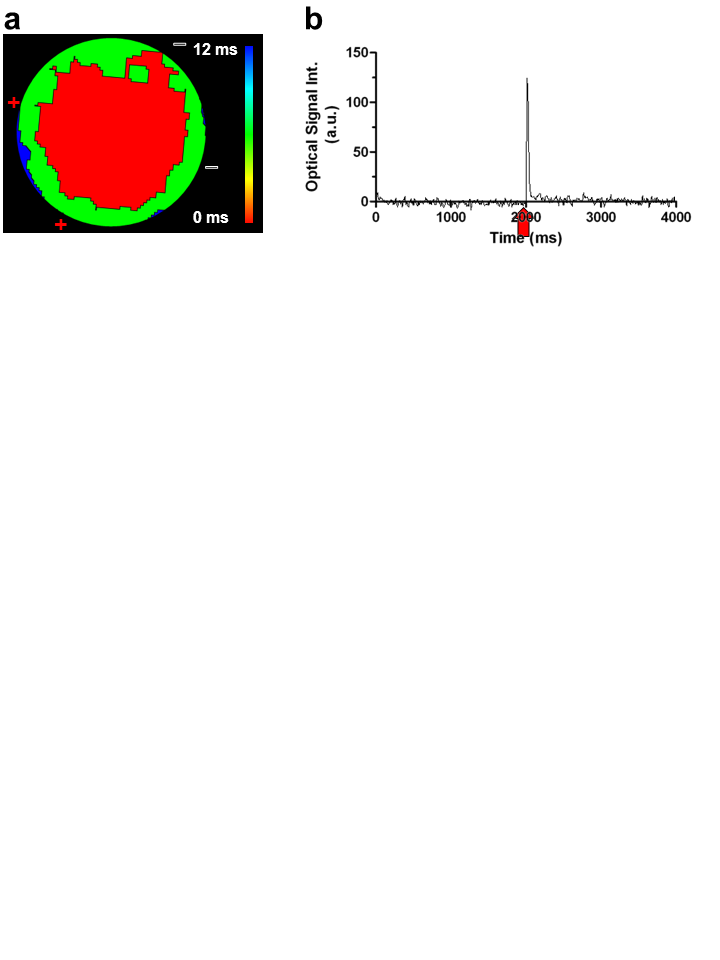
**

*Shock application without prior reentry induction.* (A) Typical activation map of a control culture subjected to a 60V shock without previous induction of reentry, showing near-simultaneous activation of the entire culture. (B) Typical single pixel recording in a culture subjected to a 60V shock without previous induction of reentry, showing a single action potential without any post-shock activity. Red arrow indicated the moment of shock application.

**Supplemental Figure 6.**

**
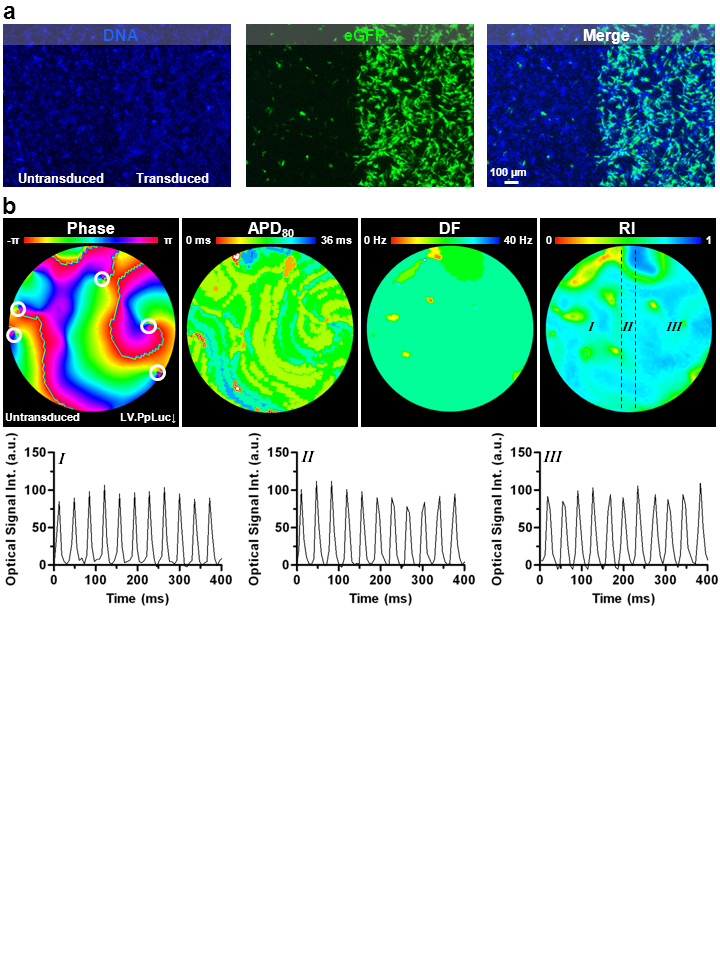
**

*Baseline electrophysiological characteristics in locally transduced aCMC cultures.* (A) Example of a fluorescent staining in a culture with the left half transduced with a control eGFP vector, and the right half left untransduced using patterned attachment showing abundant eGFP expression localized to the transduced half. (B) Typical phase, APD80, dominant frequency an regularity index map and corresponding optical signal in the untransduced area (I), the border area (II) and the transduced area (III).

**Supplemental Figure 7.**


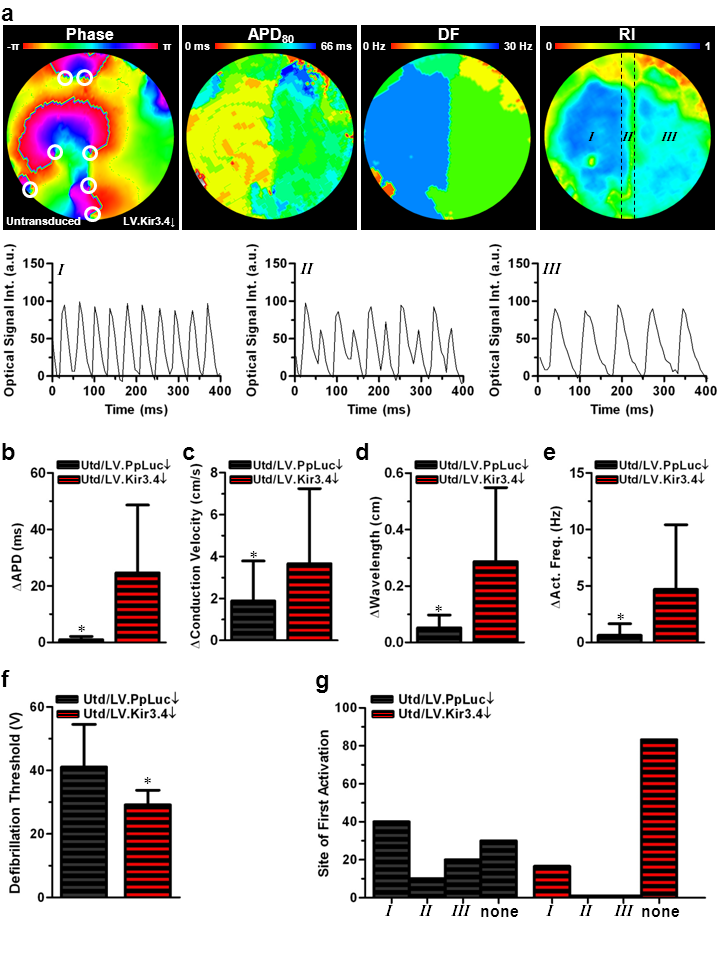


*Role of repolarization and I_K,ACh-c_-heterogeneity in post shock PS formation.* (A) Typical phase, APD80, dominant frequency an regularity index map and corresponding optical signal in the untransduced area (I), the border area (II) and the transduced area (III), showing wavebreak and APD alternans in the border area and a large APD with 2:1 conduction in the area transduced with LV.Kir3.4↓. Quantification of the difference in (B) APD_80_ (C) conduction velocity (D) wavelength and (E) activation frequency between the transduced and untransduced area in aCMC cultures locally transduced with LV.PpLuc↓ or LV.Kir3.4↓. Quantification of the difference in (F) DFT and (G) site of first activation after application of shock during fibrillation in aCMC cultures locally transduced with LV.PpLuc↓ or LV.Kir3.4↓. I: untransduced area, II: border area, III:transduced area corresponding to the areas depicted in subfigure A (upper right panel).

**Supplemental Figure 8.**

**
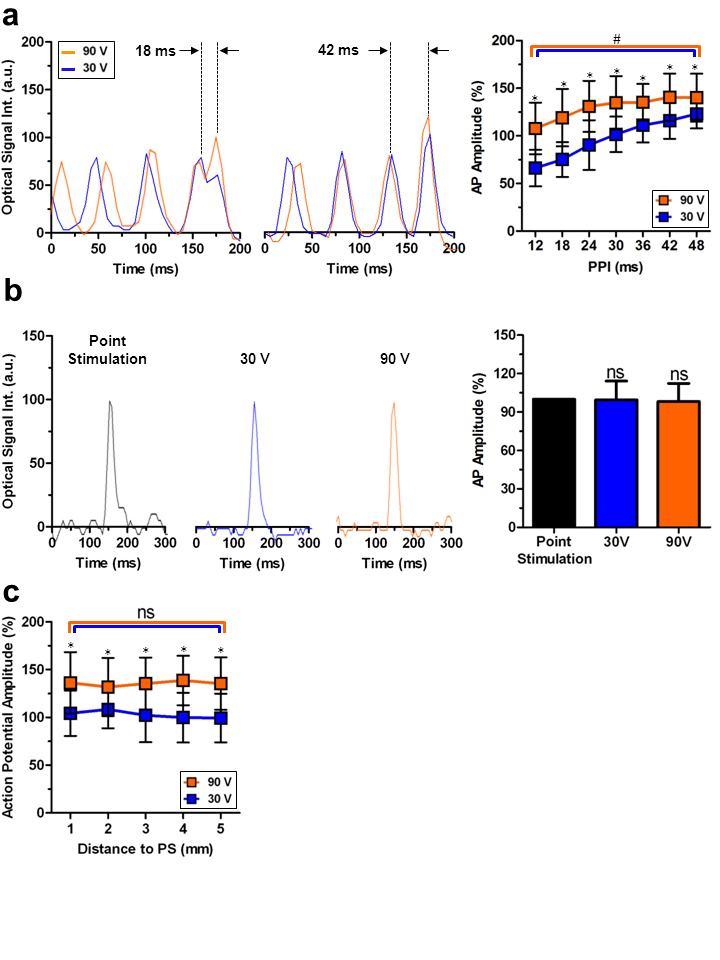
**

*Effect of shock strength on depolarization* **.** (A) Typical examples of “optical APs” during 3-4 cycles of reentrant activity followed by an electrical shock of 30 (blue) or 90 (orange) V. The timing of the electrical shocks caused a shock-induced peak in optical signal intensity 18 (left graph) or 42 (middle graph) ms after the peak of the last AP before shock delivery. On the right a quantitative analysis of the relation between post-shock action potential AP amplitude and the pre-shock peak-to-post-shock peak interval (PPI) is shown. The amplitude of the AP prior to shock delivery was set at 100%. (B) Typical examples of “optical APs” triggered by point stimulation or delivery of 30 or 90 V electrical shocks without prior induction of reentry (left panels). On the right the corresponding quantification of AP amplitude is show. The amplitude of the AP during point stimulation was set at 100%. (C) Quantification of action potential amplitude at different distances from the PS at 30V and 90V shocks. a.u.: arbitrary units. *:p<0.05 vs 30V. #:p<0.05 vs 12 ms PPI. a.u.: arbitrary units. ns: not significant vs point stimulation.

**Movie 1:**

Typical example from an optical mapping experiment in a neonatal rat atrial CMC culture exhibiting a single rotor tachyarrhythmia after burst pacing, during exposure to a high voltage shock leading to successful defibrillation (corresponding to Figure 2 in the Main Manuscript). The left panel displays the high-pass-filtered optical signal, the right panel shows the corresponding phase map progression.

**Movie 2:**

Typical example from an optical mapping experiment in a neonatal rat atrial CMC culture exhibiting a single rotor tachyarrhythmia after burst pacing, during exposure to an electric shock below DFT leading to failed defibrillation as a consequence of incomplete PS removal (corresponding to Figure 3 in the Main Manuscript). The left panel displays the high-pass-filtered optical signal, the right panel shows the corresponding phase map progression.

**Movie 3:**

Typical example from an optical mapping experiment in a neonatal rat atrial CMC culture exhibiting a single rotor tachyarrhythmia after burst pacing, during exposure to an electric shock below DFT leading to failed defibrillation as a consequence reinitiation (corresponding to Figure 4 in the Main Manuscript). The left panel displays the high-pass-filtered optical signal, the right panel shows the corresponding phase map progression.

**Reference List**

1. Bingen, B.O. et al. Atrium-Specific Kir3.x Determines Inducibility, Dynamics, and Termination of Fibrillation by Regulating Restitution-Driven Alternans. *Circulation*.**128**, 2732-2744 (2013).
2. Bingen, B.O. et al. Prolongation of minimal action potential duration in sustained fibrillation decreases complexity by transient destabilization. *Cardiovasc Res*. **97**, 161-170 (2013).
